# Innovations and Challenges in Breast Cancer Care: A Review

**DOI:** 10.3390/medicina59050957

**Published:** 2023-05-16

**Authors:** Sharat Chopra, Muskaan Khosla, Raghavan Vidya

**Affiliations:** 1 Aneurin Bevan University Health Board, The Royal Gwent Hospital, Newport NP20 2UB, UK; sharat_chopra@hotmail.com; 2 The Royal Wolverhampton NHS Trust, Wolverhampton WV10 0QP, UK; raghavan.vidya@nhs.net

**Keywords:** breast cancer, innovations, artificial intelligence, imaging, techniques, vaccines, challenges

## Abstract

Breast cancer care has seen tremendous advancements in recent years through various innovations to improve early detection, diagnosis, treatment, and survival. These innovations include advancements in imaging techniques, minimally invasive surgical techniques, targeted therapies and personalized medicine, radiation therapy, and multidisciplinary care. It is essential to recognize that challenges and limitations exist while significant advancements in breast cancer care exist. Continued research, advocacy, and efforts to address these challenges are necessary to make these innovations accessible to all patients while carefully considering and managing the ethical, social, and practical implications.

## 1. Introduction

Breast cancer is the commonest cancer among women and ranks as the second cause of cancer-related deaths in women [1]. Breast cancer care has seen significant advancements in recent years through various innovations to improve early detection, diagnosis, treatment, and survival. There have been rapid innovations in breast imaging and localization methods; genetic and genomic profiling for personalized medicine; implantable devices; and integration of artificial intelligence and deep learning models for image analysis, image-guided surgery, surgical planning, and prediction of treatment outcomes. Circumstances created by the COVID-19 pandemic have led to increased telemedicine use and have modified follow-up protocols and radiotherapy delivery methods. Additionally, with the use of more advanced prognostic and predictive tests, unnecessary/over-diagnosis, treatments, and hospital admissions [2,3,4] can be avoided.

While there have been significant advancements in breast cancer care, several ethical, social, and practical challenges have arisen along with these innovations.

In this review, we aim to describe the innovations as well as future prospects in breast cancer care, along with their limitations and challenges.

## 2. Materials and Methods

Peer-reviewed published scientific literature and continuing scientific studies were searched using relevant MeSH terms in the MEDLINE database for “innovations” OR “biomedical technology” AND “breast cancer” OR “breast surgery”. All levels of evidence were considered. Only articles published in the English language were reviewed.

We report innovations in breast cancer care, possible future applications based on the published literature and continuing scientific studies, and its potential limitations and ethical considerations.

## 3. Results and Discussions—Innovations in Breast Cancer Care

### 3.1. Diagnostic Innovations in Breast Imaging

#### 3.1.1. Mammography

The standard modality for screening and diagnosing breast malignancy has been mammography since its use following the Forrest Report in 1986, which concluded that mammographic breast cancer screening was effective in reducing breast cancer mortality and recommended the establishment of a national breast cancer screening program in the UK for women aged 50 to 64 years [5]. This cornerstone imaging technique has undergone several advancements, which is now developing into 3D mammograms or digital breast tomosynthesis (DBT). This mammography technique captures multiple breast images from different angles to create a three-dimensional image of the breast tissue using enhanced software. It thus allows for a more detailed and comprehensive view of the breast compared to traditional 2D mammography, which captures a single image. It has been demonstrated to improve breast cancer detection rates, particularly in women with dense breast tissue. Images generated by DBT allow for the evaluation of abnormal findings, such as masses or calcifications, by providing a more detailed view of their shape, size, and location within the breast tissue. However, it has increased radiation exposure compared to 2D mammography and potentially higher costs [6].

In addition, contrast-enhanced mammography (CEM) is a newly emerging tool in breast radiology which uses radioiodine/contrast material to assess breast neovascularity and provides both anatomical and local changes in the breast. Some trials have now looked into the role of CEM as a tool for screening high-risk younger patients. This has been hailed as an alternative to breast MRI and is cheaper but has a higher radiation dosage [7].

#### 3.1.2. Ultrasound Elastography

The role of ultrasound elastography has now been extended into identifying breast lesions. It uses ultrasound waves to generate tissue displacement or deformation images in response to external mechanical compression or vibration. These images are then analysed to assess the stiffness or elasticity of the breast tissue. Using a shear wave or strain wave pattern, one can differentiate a benign lesion from a malignant one. These techniques complement each other and work on the basic principle that focal breast malignant lesions are stiffer than benign lesions [8].

Guidelines recommending the use of elastography for characterizing breast lesions have been published by the European Federation of Societies for Ultrasound in Medicine (EFSUMB) and the World Federation of Ultrasound in Medicine and Biology (WFUMB) [9]. Both these guidelines recommend the addition of elastography to conventional ultrasound to improve the characterization of breast lesions as benign or malignant. Although a relatively newer technique, it has been used in other organs for assessment, including the thyroid, prostate, and liver.

#### 3.1.3. Newer Ultrasound Localization Techniques

Breast localization techniques have been known to guide surgical excisions for non-palpable or occult breast lesions using clips and wires. However, more sophisticated localization techniques have been developed, which can be deployed in advance and cause minimal tissue trauma or migration issues.

Increasing use of breast conservation surgery, especially after downstaging following neoadjuvant chemotherapy (NACT), has led to an increase in the use of novel techniques such as Magseed^®^, Radiofrequency Iodine seed (RFID) localization, Savi Scout^®^, and Pintuition^®^ to assist in precisely excising tissue of interest in theatre; this coupled with the use of 3D intraoperative X-rays have greatly helped to reduce breast margin re-excision rates and improved cosmetic outcomes, leading to better patient satisfaction and reduced readmission rates. A significant advantage is the ease of inserting these localizers under local anaesthesia in outpatient settings under ultrasound guidance, and this can be kept in the breast for six months, which can help in surgical scheduling and resource optimization [10,11,12,13].

#### 3.1.4. Outpatient Vacuum-Assisted Biopsy and Excision of Breast lesions (VAB/VAE)

The management of breast lesions with uncertain malignant potential, often called B3 lesions, is slightly complex. These lesions include lesions such as flat columnar cell atypia, atypical ductal hyperplasia (ADH), atypical intraductal epithelial proliferation (AIDEP), radial scar, papillary lesions, and lobular carcinoma in situ (LCIS) [14]. These lesions would often be removed with open surgical excision using a wire as a localizer in the past. This management has now changed to an outpatient procedure with vacuum-assisted biopsy or excision (VAB/VAE), according to the Joint Consensus Conference in Zurich in 2018 [15]. VAB/VAE obtain a larger volume of tissue equivalent to a small-wide local excision while retaining the same diagnostic accuracy as open surgery [16,17] with an overall risk for malignancy of 9.9–35.1% after total resection [18].

### 3.2. Diagnostic Pathology Innovations

#### 3.2.1. Gene Profiling of Breast Cancer

According to DNA gene profiling, breast cancer can be divided into four subtypes: Luminal A, Luminal B, HER2 enriched, and Triple negative. Multiple studies have shown that the subtypes of ER+ tumours, luminal A and B, have two distinct clinical courses. Of all breast cancer subtypes, luminal A tumours have the best prognosis, whereas luminal B, HER2-enriched, and basal subtypes are associated with poorer clinical outcomes [19]. Patients with luminal B tumours have significantly shorter overall survival (OS) and disease-free survival (DFS) times than those with luminal A breast cancer.

#### 3.2.2. HER2 Receptor Profiling

The human epidermal growth factor receptor 2 (HER2), or HER2/neu and ERBB2, encodes a transmembrane tyrosine kinase receptor that binds to its extracellular signal, initiating a cascade that mediates cell proliferation, differentiation, and survival. Between 12% and 20% of all breast cancers overexpress the HER2 protein [20] and/or have HER2 gene amplification, which results in aggressive tumour growth and is associated with poor clinical outcomes. The development of anti-HER2 therapy for women with early and advanced HER2+ breast cancer is regarded as one of the most influential successes in treating breast cancer. The commonly used anti-HER2 treatment is trastuzumab (brand name Herceptin, Genentech, San Francisco, CA, USA), and trials have shown other anti-HER2 agents, such as pertuzumab, neratinib, lapatinib, and T-DM1, to be effective. In 2005, the first trials performed in patients with operable HER2+ disease comparing trastuzumab plus chemotherapy to chemotherapy alone showed improvement in DFS and a 33% reduction in the risk of death in patients who received trastuzumab [21]. Other trials, such as CLEOPATRA [22], EMILIA [23], and TH3RESA [24], have shown HER2 targeted treatment to have a good progression-free survival (PFS).

### 3.3. Management

#### 3.3.1. Role of Oncoplastic Multidisciplinary Team Meeting (MDT)

According to the Association of Breast Surgeons (ABS) and the British Association of Plastic, Reconstructive and Aesthetic Surgeons guidelines (BAPRAS), oncoplastic breast surgery should be considered in all patients with breast cancer [25].

These discussions include volume replacement techniques, such as level 2 oncoplastic procedures and perforator flaps, and discussing the role of other reconstruction methods, such as implant-based or autologous flaps following mastectomy, as an immediate or delayed procedure [26].

#### 3.3.2. Breast Implant Registry

Following the publication of Keogh’s Review of Regulations of Cosmetic Interventions in the United Kingdom, the Breast and Cosmetic Implant Registry (BCIR) was opened in 2016 [27]. The registry records the details of any individual who has breast implant surgery for any reason and can now be traced in case of a product recall or safety concerns relating to a specific type of implant. It also allows the identification of possible trends and complications relating to a specific type of implant [28].

#### 3.3.3. Surgical Considerations

Implementing the Enhanced Recovery Programme in breast surgery has revolutionized breast surgery and improved its outcomes [29]. Using a perforator flap for reconstruction and infiltration of local anaesthesia for flap harvesting, techniques such as quilting to reduce dead space and using Tranexamic acid to reduce hematoma/bruising, are among various strategies that can reduce complication rates. The reduced dependence on opiates in breast surgery has also facilitated early discharge, thereby performing most procedures as day-cases [30].

The use of novel oncoplastic techniques to reduce mastectomy rates wherever possible has provided good cosmetic outcomes and has been the mainstay of surgical treatment for breast cancer recently. The use of techniques such as mammoplasties and local advancement perforator flaps such as lateral thoracic artery perforator (LTAP), lateral intercostal arteries (LiCAP), anterior intercostal arteries (AiCAP), thoracodorsal artery perforator (TDAP), and medial intercostal arteries (MiCAP) have all now become new standards in oncoplastic breast surgery, providing patients with more options for treatment and enabling breast conservation [31,32].

In patients where breast conservation is not an option, mastectomy is indicated, such as high-risk/BRCA genetic patients, and the choice of breast reconstruction is offered where feasible. Newer techniques, such as Prepectoral breast reconstruction using Acellular Dermal Matrix [33,34], such as Braxon^®^ and Verita, can provide immediate reconstruction with an implant. This has enabled surgeons to perform the procedure in the prepectoral space without disrupting the pectoralis muscle, which causes less post-operative pain and shoulder problems and provides good cosmetic outcomes. However, with the increased use of implants and adoption of newer cohesive breast implants, the risks of breast implant illness and low-grade anaplastic lymphoma (BIA-ALCL), with an estimated risk of 1:25,000 to 30,000 have now been added to the list of complications with the use of breast implants. These patients are informed about this while deciding on surgery [35,36].

With the increased use of breast reconstruction, modifications in the management of implant handling during surgery to reduce surgical site infection complications have also become paramount. Using theatre checklists for implants has become a standard of care in hospitals carrying out implant-based reconstruction [37].

#### 3.3.4. Oncological Considerations

##### Radiotherapy

During the COVID-19 pandemic, the management of patients with breast cancer who needed radiotherapy changed significantly. Patients who needed radiotherapy were offered a Fast Forward regime. It was established that treating patients with a 26 Gy in five fractions regimen over one week was non-inferior to the standard of 40 Gy in 15 fractions over three weeks for local tumour control. It was safe in terms of normal tissue effects for up to 5 years for patients prescribed local adjuvant radiotherapy after primary surgery for early-stage breast cancer [38].

##### Chemotherapy and Pre-optimization Exercise Prescription

There is emerging evidence that exercise therapy following breast cancer treatment significantly reduces the chances of recurrence and mortality among breast cancer patients and improves psychological well-being [39].

A meta-analysis of six studies looking at 12,108 breast cancer patients concluded that post-diagnosis physical activity reduced breast cancer deaths by nearly 34% and all-cause mortality by 41% [40]. However, this gain was mainly seen in women with oestrogen receptor-positive (ER) tumours. Patients with ER-negative tumours had no significant decrease in breast cancer-related mortality.

##### Chemotherapy and Immunotherapy

A combination of immunotherapy and chemotherapy has recently emerged as a novel treatment option, with encouraging results observed with the combination of immune checkpoint blockade with diverse biological agents, including anti-HER2 agents, cyclin-dependent kinase (CDK) 4/6 inhibitors, and PARP inhibitors. Currently, three selective CDK4/6 inhibitors (palbociclib, ribociclib, abemaciclib) have been approved by both the Food and Drug Administration (FDA) and the European Medicines Agency (EMA) for treating HR-positive, HER2-negative metastatic breast cancer [41]. Treatment with CDK4/6i in combination with endocrine therapy is generally safe and well tolerated. Haematological toxicity is commonly seen with all three inhibitors, but some haematological adverse events (AEs) are more frequent with palbociclib and ribociclib rather than abemaciclib [42]. Toxicities are easily treatable and can be managed with dose adjustment and supportive care.

### 3.4. Future of Breast Cancer Treatment

#### 3.4.1. Role of Artificial Intelligence (AI)

The evolution of AI in breast surgery had progressed from the early stages, when researchers and clinicians started exploring the potential of AI in analysing medical images, such as mammograms, to aid in breast cancer diagnosis and screening. Basic machine learning algorithms were used for image analysis and feature extraction, but the applications were limited. As technology advanced, more sophisticated deep learning algorithms, such as convolutional neural networks (CNNs), showed promise in detecting breast lesions and classifying breast cancer subtypes more accurately. These advances led to more applications of AI in breast surgery, including image-guided surgery, surgical planning, and prediction of treatment outcomes. Currently, numerous studies and clinical trials are exploring the use of AI in various aspects of breast surgery, including image analysis, surgical planning, decision support, and prediction of patient outcomes. AI-powered tools, such as computer-aided detection (CAD) systems, are increasingly used in clinical practice to assist radiologists in breast cancer screening and diagnosis. Virtual reality (VR) and augmented reality (AR) technologies are also being utilized to enhance surgical visualization and training [3,4].

The ongoing research and development pave the way for the availability in the near future of automated histopathology analysis, prediction of treatment response, and patient-specific treatment planning and optimized surgical approaches.

To resurface from the suspension of breast cancer screening during the COVID-19 infection, various centres across the United Kingdom have opted to participate in or conduct clinical trials into using AI as a screening aid for detecting breast cancer. One such centre in England, Leeds, recently announced the use of AI software as a LIBRA study [43]. The aim is to generate evidence for AI’s benefits and investigate if it could increase detection rates, reduce patient recalls, and ease workforce pressures.

Reliance on AI will likely proceed through stages. It will involve careful attention to mitigate its limitations and challenges around patient privacy, accountability, bias, informed consent, and economic and social justice [44].

Similarly, in digital pathology, transforming histopathology slides into digital images using whole-slide scanners and subsequent analysis of these digitized images by using AI appears promising. It can help speed up the diagnostic process with a limited workforce [45].

#### 3.4.2. Breast Cancer Vaccines

The role of the vaccine in breast cancer appears promising and is based on the hypothesis of immunological response via either T or B cells to destroy cancer cells. There have been several trials, but none have been promising until recently. Various types of vaccines are under trial, including peptide-based, DNA-based, tumour cell antigen-based, and carbohydrate-based vaccines [46]. Clinical trials evaluating breast cancer vaccines have provided limited evidence of clinical benefits despite the successful induction of immune responses. A potential explanation for negative results to date is that the effective anti-tumour immunity stimulated by vaccines is not long-lasting enough to produce significant benefits in survival. That the anti-tumour immune response fades so early may be attributed to the following factors: suboptimal vaccine formulations, the immune tolerance developed to specific tumour antigens, and the immune-suppressive microenvironment.

#### 3.4.3. Role of Alternative Treatment Methods

Various alternative treatment methods have been investigated recently, such as microwave ablation of breast lesions [47], cryotherapy [48,49], and laser ablation/coagulation for breast lesions. All these treatments appear very promising but are still in their infancy and can only be accomplished in trial settings. Long-term data on outcomes have rarely been reported in any studies, thus limiting its use.

#### 3.4.4. Role of Ki67 Marker

A useful clinical marker for breast cancer subtype classification, prognosis, and prediction of therapeutic response. This proliferation marker has been adopted in various centres to aid breast cancer treatment. Ki67 is a valuable tool in assessing the risk of recurrence for ER-positive human epidermal growth factor receptor 2 (HER2)-negative breast cancers, where it may be considered a surrogate of molecular assays for distinguishing luminal A from luminal B breast cancer subtypes. Two important clinical trials of NETs, the Immediate Preoperative Anastrozole, Tamoxifen, or Combined with Tamoxifen (IMPACT) trial and P024, established Ki67 as the evaluation index of NETs. IMPACT compared the efficacy of NET with anastrozole, tamoxifen, and a combination of anastrozole and tamoxifen in postmenopausal women with ER-positive invasive primary breast cancer. P024 compared letrozole with tamoxifen in NET [50,51,52].

#### 3.4.5. Role of Pathological Biomarker in Breast Cancer

The use of complex algorithms and diagnostic modalities to assess predictive and prognostic biomarkers is central to quality oncology care. Advances in sample preparation, microscopy, and image analysis have enhanced immunohistochemistry (IHC) and fluorescence in situ hybridization (FISH) methods used in HER2 borderline results. Bright-field ISH methods that can be performed using standard light microscopy, such as chromogenic ISH (CISH), silver ISH (SISH), and dual ISH (a combination of CISH and SISH techniques), have also been developed to overcome the limitations of dark-field fluorescence microscopy and the lack of morphologic details associated with FISH.

Additionally, digital microscopy and image analysis technologies are becoming increasingly important tools in pathology. For example, computerized image analysis has been shown to help improve the accuracy and reliability of IHC computerized image analysis. Imaging platforms such as the Aperio ScanScope (Aperio, Vista, CA, USA) and the Definiens (Munich, Germany) Tissue Studio allow the scanning of whole glass slides to create a digital image that can be analysed using specific algorithms within a short period and can detect regions of interest and distinguishes cells and subcellular attributes within the target regions [53].

Various gene assay techniques have now been used to manage breast cancer care: one such test is the Oncotype Dx (Genomic Health, Inc., an Exact Sciences Corporation, Madison, WI, USA) test which uses a 21 gene assay score using a quantitative reverse transcription-polymerase chain reaction (RT-PCR) assay that measures the expression of 21 genes (16 cancer-related genes and 5 reference genes that serve as internal controls). The results tell us how likely a cancer is to grow and respond to treatment. Therefore, the Oncotype DX Breast Recurrence Score Test is both a prognostic test since it provides more information about how likely (or unlikely) the breast cancer is to come back and a predictive test since it predicts the likelihood of benefit from chemotherapy or radiation therapy treatment [54]. The role of pathologists in breast cancer trials has changed considerably with a better understanding of the molecular concepts underlying the aetiology of breast cancer and the advent of targeted therapeutics and personalized medicine. Pathologists are now involved in various stages of clinical trials, from pretrial/eligibility screening to determining response to therapy. Pathologists also have a critical role in assessing the primary endpoints. The pathologic complete response (pCR), defined as the absence of residual disease after surgery, is a common endpoint in neoadjuvant trials and is assessed by the pathologist. Pathologists also play a critical role in determining the effect of biomarkers or molecular alterations on response to therapy and in monitoring the markers of response to evaluate the effectiveness of treatment, such as the role of circulating tumour cells (CTCs) in the blood, which can be an early marker of response to therapy [55].

#### 3.4.6. Proton Therapy in Breast Cancer

Radiotherapy is often used to treat breast cancer. Still, it has its limitations, such as cardiopulmonary side effects and difficult-to-treat hard-to-reach areas such as internal mammary lymph nodes, which lie close to the heart and lungs [56]. With improved technologies, precise delivery of radiotherapy has been developed to improve outcomes. With the emergence of proton beam therapy (PBT), PBT reduces nontarget average tissue exposure and may improve target coverage of difficult-to-treat areas such as the internal mammary nodes. With conventional X-rays, the radiation dose falls off gradually with depth, resulting in collateral exposure of normal tissues both proximal and distal to the targeted disease. In contrast, in PBT, the protons stop at a well-defined depth, and the dose deposition of protons reaches a maximum near the end of the proton range, resulting in less dose deposition in normal tissues, both proximal and distal to the target tissue. All this would help reduce cardiotoxic effects and complications such as radiation pneumonitis, especially in smokers. PBT is also associated with reduced skin, muscle, and bone exposure. With the heightened importance of sparing previously irradiated tissue and the high level of conformality that PBT affords, PBT is an attractive therapeutic modality for investigating patients with re-irradiation indications. A recent Radiation Therapy Oncology Group (RTOG) Phase II trial examining photon partial breast reirradiation after repeat lumpectomy for in-breast recurrence following previous whole breast irradiation showed excellent local control with acceptable early toxicity [57]. With the use of PBT, there are logistical and technical challenges, including omitting coverage of the posterolateral supraclavicular fossa (posterior triangle), which can result in microscopic disease being left untreated and can cause recurrence or metastasis. Protons are more sensitive to some intrafraction and intrafraction setup errors and changes in target volume over the course of treatment. These uncertainties can result because of inflammation of the chest wall post-surgery or the presence of breast implants or fluid collection, which may alter the dose. Lastly, the higher delivery cost compared with conventional photon techniques is also a major deterrent as it requires increased up-front capital investment [58].

### 3.5. Challenges

While there have been significant advancements in breast cancer care, several challenges have arisen along with these innovations. Some of the difficulties associated with these advancements include [2,3,4]:

*Cost and Accessibility:* Many innovative technologies and treatments in breast cancer care can be expensive, making them less accessible for some patients due to financial constraints or lack of insurance coverage. This can create disparities in access to care, with some patients unable to benefit from the latest advancements due to financial limitations.

*Ethical and Social Implications:* As personalized medicine and genomic testing become more prevalent in breast cancer care, ethical and social implications can arise, such as concerns about genetic privacy, discrimination based on genetic information, and potential psychological impacts of knowing one’s genetic predisposition to breast cancer. These challenges require careful consideration of ethical and social implications to protect patient’s rights and well-being.

*Integration and Implementation:* Integrating and implementing new technologies and treatments into routine clinical practice can be complex and challenging. Healthcare providers may have a learning curve to become proficient in using new technologies and logistical challenges regarding equipment, training, and infrastructure needed for widespread adoption.

*Evidence-based Decision Making:* While advancements in breast cancer care have brought about new treatment options, there may be limited evidence regarding their long-term safety and efficacy. This can make it challenging for healthcare providers and patients to make informed decisions about the best treatment approach, particularly in rapidly evolving fields such as immunotherapy and targeted therapies.

*Patient Education and Informed Consent:* Patient education and informed consent become crucial with the increasing complexity of breast cancer care. Ensuring that patients are adequately informed and able to make informed decisions can be challenging in the context of rapidly evolving advancements. Patients must understand different treatment options’ risks, benefits, limitations, and potential implications of genetic testing and personalized medicine.

*Health Disparities:* Despite the advancements in breast cancer care, there may be disparities in access to these innovations, particularly among underserved populations and minority communities. Addressing health disparities and ensuring equitable access to advancements in breast cancer care remains a challenge that requires attention and action.

## 4. Conclusions

We believe that through our paper, we can highlight the role of various innovations aimed at improving the ease and precision of detection, personalizing treatment, predicting response to breast care treatment, and improving overall survival. Novel advancements, however, come with newer and unique limitations and challenges. Continued research, advocacy, and bold efforts are essential to address these challenges to make these innovations accessible to all patients in an ethical, equitable, and safe environment.

## Data Availability

Not applicable.

## References

[1] Breast Cancer UK. https://www.breastcanceruk.org.uk/about-breast-cancer/facts-figures-and-qas/facts-and-figures/.

[2] Vidya R., Leff D.R., Green M., McIntosh A.S., John E.S., Kirwan C.C., Romics L., I Cutress R., Potter S., Carmichael A. (2021). Innovations for the future of breast surgery. Br. J. Surg..

[3] Soh C.L., Shah V., Rad A.A., Vardanyan R., Zubarevich A., Torabi S., Weymann A., Miller G., Malawana J. (2022). Present and future of machine learning in breast surgery: Systematic review. Br. J. Surg..

[4] Romeo V., Accardo G., Perillo T., Basso L., Garbino N., Nicolai E., Maurea S., Salvatore M. (2021). Assessment and Prediction of Response to Neoadjuvant Chemotherapy in Breast Cancer: A Comparison of Imaging Modalities and Future Perspectives. Cancers.

[5] Forrest A.P.M. (1986). Breast Cancer Screening: Report to the Health Ministers of England, Wales, Scotland and Northern Ireland.

[6] Kulkarni S., Freitas V., Muradali D. (2022). Digital Breast Tomosynthesis: Potential Benefits in Routine Clinical Practice. Can. Assoc. Radiol. J..

[7] Jochelson M.S., Lobbes M.B.I. (2021). Contrast-enhanced Mammography: State of the Art. Radiology.

[8] Barr R.G. (2018). The Role of Sonoelastography in Breast Lesions. Semin. Ultrasound CT MRI.

[9] Shiina T., Nightingale K.R., Palmeri M.L., Hall T.J., Bamber J.C., Barr R.G., Castera L., Choi B.I., Chou Y.-H., Cosgrove D. (2015). WFUMB guidelines and recommendations for clinical use of ultrasound elastography: Part 1: Basic principles and terminology. Ultrasound Med. Biol..

[10] Kabeer K.K., Gowda S.M., Mohd-Isa Z., Thomas M.J.R., Gopalan V., Jafferbhoy S., Soumian S., Narayanan S., Kirby R., Marla S. (2022). An Audit on Oncological Safety with Magseed Localised Breast Conserving Surgery. Indian J. Surg. Oncol..

[11] Davey M.G., O’Donnell J.P.M., Boland M.R., Ryan É.J., Walsh S.R., Kerin M.J., Lowery A.J. (2022). Optimal localisation strategies for non-palpable breast cancers—A network meta-analysis of randomised controlled trials. Breast.

[12] Kasem I., Mokbel K. (2020). Savi Scout^®^ Radar Localisation of Non-palpable Breast Lesions: Systematic Review and Pooled Analysis of 842 Cases. Anticancer Res..

[13] Dave R.V., Barrett E., Morgan J., Chandarana M., Elgammal S., Barnes N., Sami A., Masudi T., Down S., Holcombe C. (2022). Wire- and magnetic-seed-guided localisation of impalpable breast lesions: iBRA-NET localisation study. Br. J. Surg..

[14] Hoon Tan P., Ellis I., Allison K., Brogi E., Fox S.B., Lakhani S., Lazar A.J., Morris E.A., Sahin A., Salgado R. (2020). The 2019 World Health Organization classification of tumours of the breast. Histopathology.

[15] Rageth C.J., O’flynn E.A.M., Pinker K., Kubik-Huch R.A., Mundinger A., Decker T., Tausch C., Dammann F., Baltzer P.A., Fallenberg E.M. (2019). Second International Consensus Conference on lesions of uncertain malignant potential in the breast (B3 lesions). Breast Cancer Res. Treat..

[16] O’Flynn E.A., Wilson A.R., Michell M.J. (2010). Image-guided breast biopsy: State-of-the-art. Clin. Radiol..

[17] McMahon M.A., Haigh I., Chen Y., Millican-Slater R.A., Sharma N. (2020). Role of vacuum assisted excision in minimising overtreatment of ductal atypias. Eur. J. Radiol..

[18] Bianchi S., Caini S., Renne G., Cassano E., Ambrogetti D., Cattani M.G., Saguatti G., Chiaramondia M., Bellotti E., Bottiglieri R. (2011). Positive predictive value for malignancy on surgical excision of breast lesions of uncertain malignant potential (B3) diagnosed by stereotactic vacuum-assisted needle core biopsy (VANCB): A large multi-institutional study in Italy. Breast.

[19] Prat A., Pineda E., Adamo B., Galván P., Fernández A., Gaba L., Díez M., Viladot M., Arance A., Muñoz M. (2015). Clinical implications of the intrinsic molecular subtypes of breast cancer. Breast.

[20] Martínez-Sáez O., Prat A. (2021). Current and Future Management of HER2-Positive Metastatic Breast Cancer. JCO Oncol. Pract..

[21] Romond E.H., Perez E.A., Bryant J., Suman V.J., Geyer C.E., Davidson N.E., Tan-Chiu E., Martino S., Paik S., Kaufman P.A. (2005). Trastuzumab plus adjuvant chemotherapy for operable HER2-positive breast cancer. N. Engl. J. Med..

[22] Baselga J., Swain S.M. (2010). CLEOPATRA: A phase III evaluation of pertuzumab and trastuzumab for HER2-positive metastatic breast cancer. Clin. Breast Cancer.

[23] Verma S., Miles D., Gianni L., Krop I.E., Welslau M., Baselga J., Pegram M., Oh D.-Y., Diéras V., Guardino E. (2012). Trastuzumab emtansine for HER2-positive advanced breast cancer. N. Engl. J. Med..

[24] Krop I.E., Kim S.B., González-Martín A., LoRusso P.M., Ferrero J.M., Smitt M., Yu R., Leung A.C., Wildiers H., TH3RESA study collaborators (2014). Trastuzumab emtansine versus treatment of physician’s choice for pretreated HER2-positive advanced breast cancer (TH3RESA): A randomised, open-label, phase 3 trial. Lancet Oncol..

[25] Gilmour A., Cutress R., Gandhi A., Harcourt D., Little K., Mansell J., Murphy J., Pennery E., Tillett R., Vidya R. (2021). Oncoplastic breast surgery: A guide to good practice. Eur. J. Surg. Oncol..

[26] MacNeill F., Tasoulis M.K., Tan M.L.H., Karakatsanis A., Wyld L., Markopoulos C., Leidenius M., Senkus-Konefka E. (2018). The Breast and Oncoplastic Multidisciplinary Team. Breast Cancer Management for Surgeons.

[27] Keogh Poly Implant Prothèse (PIP) Breast Implants: Final Report of the Expert Group. https://assets.publishing.service.gov.uk/government/uploads/system/uploads/attachment_data/file/192028/Review_of_the_Regulation_of_Cosmetic_Interventions.pdf.

[28] Breast and Cosmetic Implant Registry (BCIR). https://digital.nhs.uk/data-and-information/clinical-audits-and-registries/breast-and-cosmetic-implant-registry.

[29] Offodile A.C., Gu C., Boukovalas S., Coroneos C.J., Chatterjee A., Largo R.D., Butler C. (2019). Enhanced recovery after surgery (ERAS) pathways in breast reconstruction: Systematic review and meta-analysis of the literature. Breast Cancer Res. Treat..

[30] Batdorf N.J., Lemaine V., Lovely J.K., Ballman K.V., Goede W.J., Martinez-Jorge J., Booth-Kowalczyk A.L., Grubbs P.L., Bungum L.D., Saint-Cyr M. (2015). Enhanced recovery after surgery in microvascular breast reconstruction. J. Plast. Reconstr. Aesthetic Surg..

[31] McCulley S.J., Schaverien M.V., Tan V.K., Macmillan R.D. (2015). Lateral thoracic artery perforator (LTAP) flap in partial breast reconstruction. J. Plast. Reconstr. Aesthetic Surg..

[32] Hamdi M., Van Landuyt K., Monstrey S., Blondeel P. (2004). Pedicled perforator flaps in breast reconstruction: A new concept. Br. J. Plast. Surg..

[33] Chopra S., Rehnke R.D., Vidya R. (2021). Implant-based Prepectoral Breast Reconstruction: The Importance of Oncoplastic Plane, its Blood Supply and Assessment Methods. World J. Plast. Surg..

[34] Chopra S., Al-Ishaq Z., Vidya R. (2021). The Journey of Prepectoral Breast Reconstruction through Time. World J. Plast. Surg..

[35] Nelson J.A.M., Dabic S.M., Mehrara B.J., Cordeiro P.G., Disa J.J., Pusic A.L.M., Matros E.M., Dayan J.H., Allen R.J.J., Coriddi M. (2020). Breast Implant-associated Anaplastic Large Cell Lymphoma Incidence: Determining an Accurate Risk. Ann. Surg..

[36] Kaplan J., Rohrich R. (2021). Breast implant illness: A topic in review. Gland. Surg..

[37] Barr S., Topps A., Barnes N., Henderson J., Hignett S., Teasdale R., McKenna A., Harvey J., Kirwan C. (2016). Infection prevention in breast implant surgery-A review of the surgical evidence, guidelines and a checklist. Eur. J. Surg. Oncol..

[38] Murray Brunt A., Haviland J.S., Wheatley D.A., Sydenham M.A., Alhasso A., Bloomfield D.J., Chan C., Churn M., Cleator S., Coles C.E. (2020). Hypofractionated breast radiotherapy for 1 week versus 3 weeks (FAST-Forward): 5-year efficacy and late normal tissue effects results from a multicentre, non-inferiority, randomised, phase 3 trial. Lancet.

[39] Cannioto R., Hutson A., Dighe S., McCann W., E McCann S., Zirpoli G.R., Barlow W., Kelly K.M., A DeNysschen C., Hershman D.L. (2021). Physical Activity Before, During, and After Chemotherapy for High-Risk Breast Cancer: Relationships With Survival. J. Natl. Cancer Inst..

[40] Ibrahim E.M., Al-Homaidh A. (2021). Physical activity and survival after breast cancer diagnosis: Meta-analysis of published studies. Med. Oncol..

[41] Miglietta F., Cona M.S., Dieci M.V., Guarneri V., La Verde N. (2020). An overview of immune checkpoint inhibitors in breast cancer. Explor. Target. Antitumor Ther..

[42] Cardoso F., Senkus E., Costa A., Papadopoulos E., Aapro M., André F., Harbeck N., Aguilar Lopez B., Barrios C.H., Bergh J. (2018). 4th ESO-ESMO International Consensus Guidelines for Advanced Breast Cancer (ABC 4)†. Ann. Oncol..

[43] Leeds Teaching Hospitals Trials AI Software in Breast Cancer Screening. https://www.digitalhealth.net/2023/03/leeds-teaching-hospitals-trials-ai-software-in-breast-cancer-screening/.

[44] Morgan M.B., Mates J.L. (2021). Applications of Artificial Intelligence in Breast Imaging. Radiol. Clin. N. Am..

[45] Ibrahim A., Gamble P., Jaroensri R., Abdelsamea M.M., Mermel C.H., Chen P.C., Rakha E.A. (2020). Artificial intelligence in digital breast pathology: Techniques and applications. Breast.

[46] Zhu S.Y., Yu K.D. (2022). Breast Cancer Vaccines: Disappointing or Promising?. Front. Immunol..

[47] Zhou W., Yu M., Pan H., Qiu W., Wang H., Qian M., Che N., Zhang K., Mao X., Li L. (2021). Microwave ablation induces Th1-type immune response with activation of ICOS pathway in early-stage breast cancer. J. Immunother. Cancer..

[48] Palussiere J., Henriques C., Mauriac L., Asad-Syed M., Valentin F., Brouste V., Mathoulin-Pélissier S., De Lara C.T., Debled M. (2012). Radiofrequency ablation as a substitute for surgery in elderly patients with nonresected breast cancer: Pilot study with long-term outcomes. Radiology.

[49] Cazzato R.L., De Lara C.T., Buy X., Ferron S., Hurtevent G., Fournier M., Debled M., Palussière J. (2015). Single-Centre Experience with Percutaneous Cryoablation of Breast Cancer in 23 Consecutive Non-surgical Patients. Cardiovasc. Interv. Radiol..

[50] Smith I.E., Dowsett M., Ebbs S.R., Dixon J.M., Skene A., Blohmer J.-U., Ashley S.E., Francis S., Boeddinghaus I., Walsh G. (2005). Neoadjuvant Treatment of Postmenopausal Breast Cancer With Anastrozole, Tamoxifen, or Both in Combination: The Immediate Preoperative Anastrozole, Tamoxifen, or Combined With Tamoxifen (IMPACT) Multicenter Double-Blind Randomized Trial. J. Clin. Oncol..

[51] Ellis M.J., Miller W.R., Tao Y., Evans D.B., Ross H.A.C., Miki Y., Suzuki T., Sasano H. (2009). Aromatase Expression and Outcomes in the P024 Neoadjuvant Endocrine Therapy Trial. Breast Cancer Res. Treat..

[52] Dowsett M., Smith I.E., Ebbs S.R., Dixon J.M., Skene A., Griffith C., Boeddinghaus I., Salter J., Detre S., Hills M. (2005). Short-Term Changes in Ki-67 During Neoadjuvant Treatment of Primary Breast Cancer With Anastrozole or Tamoxifen Alone or Combined Correlate With Recurrence-Free Survival. Clin. Cancer Res. An. Off. J. Am. Assoc. Cancer Res..

[53] Lloyd M.C., Allam-Nandyala P., Purohit C.N., Burke N., Coppola D., Bui M.M. (2010). Using image analysis as a tool for assessment of prognostic and predictive biomarkers for breast cancer: How reliable is it?. J. Pathol. Inform..

[54] Allison K.H. (2021). Prognostic and predictive parameters in breast pathology: A pathologist’s primer. Mod. Pathol..

[55] Krol I., Schwab F.D., Carbone R., Ritter M., Picocci S., De Marni M.L., Stepien G., Franchi G.M., Zanardi A., Rissoglio M.D. (2021). Detection of clustered circulating tumour cells in early breast cancer. Br. J. Cancer.

[56] Lin L.L., Vennarini S., Dimofte A., Ravanelli D., Shillington K., Batra S., Tochner Z., Both S., Freedman G. (2015). Proton beam versus photon beam dose to the heart and left anterior descending artery for left-sided breast cancer. Acta Oncol..

[57] Arthur D., Moughan J., Kuerer H., Haffty B., Cuttino L., Todor D., Simone N., Hayes S., Woodward W., McCormick B. (2016). NRG Oncology/RTOG 1014: 3-year efficacy report from a Phase II study of repeat breast preserving surgery and 3D conformal partial breast re-irradiation (PBrI) for in-breast recurrence. Int. J. Radiat. Oncol. Biol. Phys..

[58] Lievens Y., Pijls-Johannesma M. (2013). Health economic controversy and cost-effectiveness of proton therapy. Semin. Radiat. Oncol..

